# Hippocampal Volumetric Changes in Astronauts Following a Mission in the International Space Station

**DOI:** 10.3390/neurosci6030070

**Published:** 2025-07-25

**Authors:** Shafaq Batool, Tejdeep Jaswal, Ford Burles, Giuseppe Iaria

**Affiliations:** 1 Canadian Space Health Research Network, Calgary, AB T2N 1N4, Canada; shafaq.batool@ucalgary.ca (S.B.); tejdeep.jaswal@ucalgary.ca (T.J.); cfburles@ucalgary.ca (F.B.); 2 NeuroLab, Department of Psychology, Hotchkiss Brain Institute, Alberta Children’s Hospital Research Institute, University of Calgary, Calgary, AB T2N 1N4, Canada

**Keywords:** brain, confinement, isolation, NASA, neuroplasticity, space

## Abstract

(1) Background: Evidence from non-human animal and spaceflight analog studies have suggested that traveling to outer space could have a significant impact on the structural properties of the hippocampus, a brain region within the medial temporal lobe that is critical for learning and memory. Here, we tested this hypothesis in a group of astronauts who participated in a six-month mission in the International Space Station (ISS). (2) Methods: We collected magnetic resonance imaging (MRI) scans from a sample of 17 (9 males, 8 females) astronauts before and after the ISS mission, and calculated percent gray matter volume changes in the whole hippocampus and its (anterior, body, and posterior) subregions in both hemispheres. (3) Following the six-month mission in the ISS, we found a significantly decreased volume in the whole left hippocampus; in addition, when looking at subregions separately, we detected a significantly decreased volume in the anterior subregion of the left hippocampus and the body subregion of the right hippocampus. We also found a significantly decreased volume in the whole right hippocampus of male astronauts as compared to female astronauts. (4) Conclusions: This study, providing the very first evidence of hippocampal volumetric changes in astronauts following a six-month mission to the ISS, could have significant implications for cognitive performance during future long-duration spaceflights.

## 1. Introduction

It may seem glamorous to travel to outer space. However, the experience brings with it a range of profound physiological and psychological challenges. In fact, during a spaceflight, astronauts are routinely exposed to a unique combination of environmental stressors—including microgravity, cosmic radiation, isolation, and confinement—which collectively strain multiple systems in the body. Among the most vulnerable is the central nervous system [1]. From a neurological perspective, mounting evidence indicates that spaceflight can exert significant effects on the brain, with some studies pointing to potential long-term consequences for both structural and functional properties [2,3].

One brain region that has drawn increasing scientific concern related to space travel is the hippocampus—a bilateral structure in the medial temporal lobe that plays a critical role in learning, memory consolidation, and spatial navigation [4]. The hippocampus is also one of the most plastic and stress-sensitive regions in the adult brain, known to undergo structural and functional changes in response to environmental challenges. This heightened vulnerability is thought to stem, in part, from the hippocampus’s high density of glucocorticoid receptors, making it highly reactive to elevated stress hormones such as cortisol [5]. Furthermore, it is one of the few regions in the adult brain where neurogenesis continues throughout life—particularly in the dentate gyrus—a process that is acutely sensitive to radiation, oxidative stress, and reduced environmental stimulation [6]. These mechanisms make the hippocampus especially susceptible to the unique neurophysiological and environmental stressors encountered by astronauts during a spaceflight [7].

Yet despite its centrality to cognition and its known susceptibility to stress, the hippocampus remains relatively understudied in the context of actual human spaceflight. While several studies have documented global changes in brain volume, cerebrospinal fluid distribution, and cortical architecture following long-duration missions [8], they have not examined localized structural changes in the hippocampus specifically. Past astronaut neuroimaging studies have employed whole-brain volumetric approaches or have focused on gross measures such as ventricular expansion and cortical thickness [8]. However, such methods may lack the resolution to detect more subtle, region-specific alterations; in fact, without targeted analyses using standardized anatomical protocols, changes in structures like the hippocampus—which are smaller, but functionally critical—may go unnoticed.

Currently, extensive findings from non-human animal models and spaceflight analogs provide a strong basis for hypothesizing that the hippocampus is particularly vulnerable to the combined stressors of spaceflight—and that this vulnerability may manifest as structural changes with potential cognitive consequences. Studies in rodents have shown that simulated microgravity—induced through hindlimb unloading paradigms—can reduce hippocampal neurogenesis and impair dendritic complexity, resulting in measurable deficits in spatial learning and memory [8,9,10,11]. Similarly, studies examining the effects of space-relevant radiation exposures (e.g., heavy-ion or proton radiation) have reported disrupted synaptic function, oxidative stress, and long-term impairments in hippocampus-dependent tasks in animal models [12,13,14,15,16].

Human analog environments—including long-duration bed rest, Antarctic overwintering, and underwater confinement—offer complementary insight into hippocampal vulnerability. These analogs replicate many aspects of the spaceflight experience, such as immobility, isolation, and reduced sensory input, and have consistently been associated with hippocampal volumetric reductions, disrupted functional connectivity, and altered neural activity during cognitive tasks [7,11,17,18,19]. For instance, Friedl-Werner and colleagues (2020) demonstrated that 60 days of head-down-tilt bed rest significantly altered hippocampal activity during memory encoding and retrieval, suggesting that even in the absence of radiation, extended confinement in simulated microgravity impacts hippocampal functioning [20].

By integrating findings from histological and proteomic analyses in animal models, neuroimaging in analog environments, and behavioral assessments across contexts, a coherent body of evidence identifies the hippocampus as a key neural substrate affected by spaceflight. Each method contributes distinct insights—animal studies elucidate mechanisms under controlled conditions, analog environments simulate mission-relevant stressors, and astronaut imaging provides direct evidence of brain changes in flight. Together, these approaches consistently highlight the hippocampus as both structurally vulnerable and cognitively essential in the context of space exposure.

This functional importance makes hippocampal integrity essential to astronaut performance. In fact, the hippocampus supports key cognitive processes such as spatial orientation, environmental mapping, memory updating, and decision-making—all of which are indispensable during mission-critical operations in space [21,22]. Subtle damage to this region could undermine situational awareness, increase response errors, or delay adaptation to unfamiliar environments during both flight and reentry phases [23]. As such, even minor structural alterations may carry significant operational consequences.

While the hippocampus is often treated as a unified structure in neuroimaging studies, increasing evidence supports the need for segmentation into its anterior (head), body, and posterior (tail) regions to capture its functional and structural heterogeneity. Each subregion has distinct cytoarchitectural and connectivity profiles that underlie specialized cognitive functions. The anterior hippocampus exhibits stronger connectivity with the amygdala and anterior temporal lobe and is more involved in emotional processing and associative or item-based memory, whereas the posterior hippocampus is more closely linked with parietal and retrosplenial regions and is implicated in spatial navigation and contextual memory [24]. Structural MRI and resting-state fMRI studies have demonstrated that these subdivisions are not arbitrary but reflect meaningful differences in both gray matter integrity and intrinsic connectivity [25]. Moreover, the anterior–posterior axis shows differential vulnerability to aging and disease: posterior atrophy is more strongly associated with memory decline, while anterior volume loss correlates with affective symptoms in conditions like depression and early Alzheimer’s disease [26]. Therefore, segmenting the hippocampus not only provides a more accurate understanding of its role in cognition but also enhances the sensitivity of MRI-based analyses in detecting subregion-specific alterations in both healthy and clinical populations.

Despite this growing body of indirect evidence, no study to date has specifically and systematically examined the hippocampus in astronauts using high-resolution neuroimaging. Although broader analyses have identified post-flight brain volume shifts and ventricular expansion [8], the hippocampus has not yet been isolated as a region of interest—despite its essential role in memory, learning, and spatial cognition, all of which are critical to successful in-mission performance and post-mission recovery. Moreover, given the hippocampus’s role in cognitive resilience and adaptability, detecting even subtle structural changes in this region could provide an early warning sign of potential cognitive risks faced by astronauts [27].

In this study, we address this critical gap by directly evaluating hippocampal structure in astronauts who completed approximately six-month missions aboard the International Space Station (ISS). Using high-resolution structural MRI and a harmonized hippocampal segmentation protocol, we assessed pre- and post-flight hippocampal volumes to determine whether a long-duration spaceflight leads to measurable alterations in this key brain region. We hypothesized that spaceflight may have a detrimental impact on the structural integrity of the hippocampus, resulting in a significantly decreased volume as detected following the mission in the ISS.

## 2. Materials and Methods

**Figure 1:**
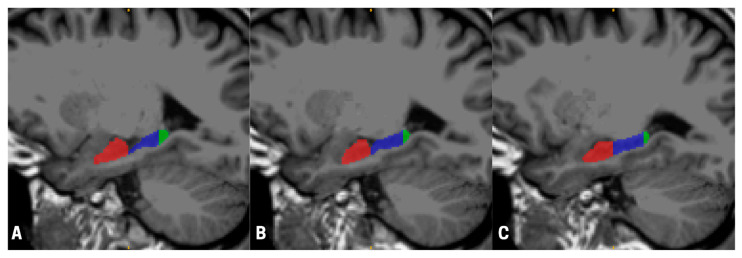
Sagittal views of a T1-weighted magnetic resonance imaging (MRI) scan illustrating hippocampal subregional segmentation across three medial-to-lateral slices (**A**–**C**). The anterior hippocampus is shown in red, the body in blue, and the posterior hippocampus in green. Segmentation was performed automatically using FreeSurfer and manually quality-checked in accordance with the Harmonized Hippocampal Protocol [29] to ensure anatomical accuracy. Visualizations were rendered using ITK-SNAP (version 0.19.2), with panels progressing laterally toward the outer edge of the skull (**A**–**C**).

We acquired magnetic resonance imaging (MRI) scans from a sample of 17 (9 males, 8 females) astronauts (mean age 45.32 years—SD 5.76 years) who participated in typical ISS missions with an average duration of 189.00 (SD = 63.18) days. Data were collected an average of 223.94 (SD = 122.23) days before launch and 12.59 (SD = 1.87) days after landing as part of the Canadian Space Agency (CSA) ‘Wayfinding’ project. The scans were performed on a 3T Siemens Verio at the League City University of Texas Medical Branch Campus using a 32-channel head coil. For each participant, before and after their space mission, we acquired a T1-weighted MPRAGE (TR = 2.3 s, TE = 2.34 ms, TI = 0.9 s, FA = 8°, sagittal acquisition with in-plane resolution of 0.9766 by 0.9766 mm, and a slice thickness of 1 mm) and a FLAIR (TR = 5 s, TE = 354 ms, TI = 1.8 s, FA = 120°, sagittal acquisition with in-plane resolution of 0.9766 by 0.9766 mm, and a slice thickness of 1 mm) sequence, collected on Syngo version B19. For each participant, we used Advanced Normalization Tools (ANTs) (ANTs v2.5.4, ANTsX ecosystem) to register the MPRAGE image to the FLAIR image, and subsequently passed this data to FreeSurfer’s recon-all pipeline. Finally, we used FreeSurfer’s hippocampal subfields, segmentHA_T2.sh, to compute hippocampal volumes [28] (see Figure 1).

We performed a manual inspection of each automatic hippocampal segmentation for quality control according to the Harmonized Hippocampal Protocol [29], and extracted volume measurements for each hippocampal subregion on both pre- and post-mission scans. Finally, we calculated the percent volume change between pre- and post-mission scans (on the whole hippocampus and subregions), used to best control for individual variability in brain volume/size between participants included in the study [20]. Finally, we calculated the percent volume changes using the following formula:

% Volume Change = [(Post-Mission Volume − Pre-Mission Volume)/Pre-Mission Volume] × 100

To test the hypothesis that structural changes are confined to the hippocampus, we also segmented and calculated the pre- and post-mission percent changes in two control brain regions, these being the amygdala [30] and the caudate nucleus [31], in both the left and right hemispheres. We used FreeSurfer’s aseg statistics for amygdala and caudate nucleus volumes.

We employed a within-subject design using one-sample *t*-tests to determine whether the percent volume change in each hippocampal subregion (left/right anterior, body, and posterior) significantly differed from zero, which served as the reference point representing no structural change from pre- to post-flight. Analyses were conducted separately by hemisphere and subregion to assess region-specific effects. To explore potential demographic influences, independent-samples *t*-tests were used to examine sex differences in percent volume change across the left and right hippocampus as well as its regions.

While our primary focus was on the hippocampus, the amygdala and caudate nucleus [30,31] were included as anatomical and functional control regions, respectively, and were analyzed using the same statistical approach to assess the specificity of observed hippocampal effects.

To evaluate whether astronauts exhibited greater hippocampal atrophy than expected for their age, we developed a normative model of hippocampal aging using population-level data from the UK Biobank (*N* = 19,793). Specifically, we used the age-stratified hippocampal volume estimates reported by Nobis and colleagues [32], which provided sex-specific normative trajectories of hippocampal volume across the adult lifespan. These data were derived from healthy adults and represent one of the most robust normative neuroanatomical reference sets available [32].

Using these values, we modeled the expected hippocampal volume decline beyond age 50 using a segmented regression (joinpoint) approach. Among four candidate models, the best-fitting model (Model 1A) was a simple linear regression with no joinpoints. This model demonstrated the lowest mean squared error (MSE = 0.979) and a statistically significant negative slope (*p* = 0.001), consistent with prior findings of steady, age-related hippocampal decline in midlife and older adulthood.

The resulting equation to estimate age-related hippocampal volume V(x) in mm^3^ was

V(x) = 7929.60 − 8.63 × x

In the equation, x is the number of years after age 50, V(x) is the expected hippocampal volume at age 50 + x, 7929.60 mm^3^ is the estimated volume at age 50, and −8.63 mm^3^/year is the predicted annual decline in volume.

Since our outcome variable was the percent volume change across approximately one year, the model was reframed to express the expected annual percent volume change due to aging at each individual’s age. For each astronaut, we first used the model to estimate their expected hippocampal volume at baseline age a:

V(a) = 7929.60 − 8.63 × (a − 50)

Then, the expected annual percent change at that age was computed by dividing the annual volume decline (−8.63 mm^3^) by the expected baseline volume for that age:

Expected annual percent change at age a = (−8.63/expected volume at age a) × 100

This provided a continuous, age-specific prediction of how much hippocampal volume would be expected to decrease in one year due to normal aging. These individualized estimates were then compared with a paired *t*-test with the observed percent changes in the left (%_lh_change) and right (%_rh_change) hippocampus and each compared to their age-specific expected values. All analyses were conducted using JASP (version 0.19.2).

## 3. Results

**Figure 2:**
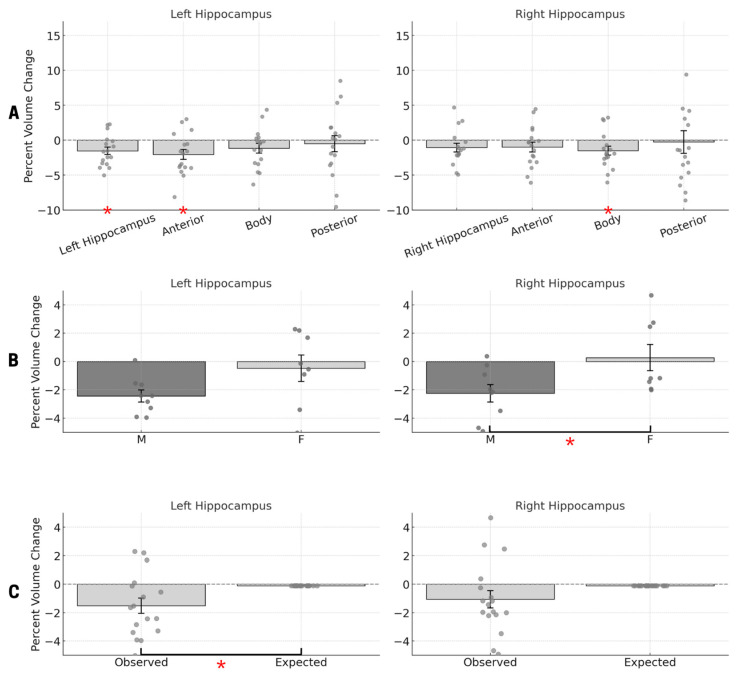
Percent volume change in the hippocampus from pre- to post-spaceflight: (**A**) Percent volume change in the left (on the left) and right hippocampus (on the right) and their subregions (anterior, body, and posterior), averaged across all participants (not stratified by sex), evaluated with a one-sample *t*-test. Significant volume decrease was observed in the left hippocampus (*p* = 0.013) and the anterior subregion (*p* = 0.012) and in the right body subregion (*p* = 0.035). (**B**) Sex differences in percent volume change for the left and right whole hippocampus. Males (dark gray) and females (light gray) are compared with an independent-samples *t*-test, with a significant sex difference observed in the right hippocampus (*p* = 0.035), where males exhibited greater volume loss than females. (**C**) Comparison of observed hippocampal volume change in astronauts with expected changes based on a modeled healthy aging population evaluated with a paired-samples *t*-test. A significant difference was observed for the left hippocampus (*p* = 0.012), indicating volume loss beyond that expected from aging alone. Asterisks (*) indicate statistically significant results at *p* < 0.05. Error bars represent standard error.

We tested significant differences in percent change for each region independently so as to compare each percent volume change against no change. Results are reported in Figure 2.

First, we found a significant mean percent volume change of −1.513 in the left hippocampus (*t*(16) = −2.812, *p* = 0.013), and no significant mean percent volume changes in the right hippocampus (%volumechange: −1.062; *t*(16) = −1.736, *p* = 0.102) (see Figure 2A). As for changes in the control regions, we found no significant differences in the left (%volumechange: −0.916; *t*(16) = −0.805, *p* = 0.433) and right (%volumechange: −0.870; *t*(16) = −0.667, *p* = 0.514) amygdala (see Supplementary Figure S1A), as well as in the left (%volumechange: 0.564; *t*(16) = 0.586, *p* = 0.566) and right (%volumechange: 0.311; *t*(16) = −0.477, *p* = 0.640) caudate nucleus (see Supplementary Figure S1A).

With respect to the anterior, midsection (body), and posterior subregions of the left hippocampus, our analyses revealed a significant percent volume change of −2.037 in the anterior subregion (*t*(16) = −2.840, *p* = 0.012), and no significant changes in the body (%volumechange: −1.158; *t*(16) = −1.701, *p* = 0.108) and posterior (%volumechange: −0.501; *t*(16) = −0.438, *p* = 0.667) subregions (see Figure 2A). In the right hippocampus, we found a significant percent volume change of −1.492 in the body subregion (*t*(16) = −2.302, *p* = 0.035), and no significant changes in the anterior (%volumechange: −0.987; *t*(16) = −1.388, *p* = 0.184) and posterior (%volumechange: −0.258; *t*(16) = −0.160, *p* = 0.875) subregions (see Figure 2A).

Lastly, we used an independent *t*-test analysis with sex as a grouping variable to explore the effect of astronauts’ sex on their hippocampal structural changes following the space mission. We found a significant volume change in the right hippocampus (*t*(15) = −2.319, *p* = 0.035), with male astronauts showing a larger decrease (−2.247) as compared to female astronauts (0.271); however, no statistically significant difference was observed in the left hippocampus (*t*(15) = −1.975, *p* = 0.067) between male (−2.435) and female (−0.476) astronauts (see Figure 2B).

We also conducted analyses to assess sex-based differences in percentage changes across hippocampal (anterior, body, and posterior) subregions in the left and right hippocampus, and found no significant difference in percent volume change between males and females (left anterior hippocampus: %volumechange of −2.988, *t*(15) = −1.453, *p* = 0.167; right anterior hippocampus: %volumechange of −2.113, *t*(15) = −1.794, *p* = 0.093; left hippocampal body: %volumechange of −1.798, *t*(15) = −0.998, *p* = 0.334; right hippocampal body: %volumechange of −2.537, *t*(15) = −1.832, *p* = 0.087; left posterior hippocampus: %volumechange of −1.729, *t*(15) = −1.150, *p* = 0.268; right posterior hippocampus: %volumechange: −2.082, *t*(15) = −1.219, *p* = 0.242) (see Supplementary Figure S1C,D).

To assess whether hippocampal volume change in astronauts exceeded the annual expected change in a healthy population, paired-samples *t*-tests were conducted for the left and right hippocampus. Results revealed a significant difference between the percent volume change in the left hippocampus and the annual expected change, with *t*(16) = −2.611 and *p* = 0.019, suggesting that the left hippocampus exhibited significantly greater atrophy than expected. The average percent change in the left hippocampus was −1.513% (SD = 2.218), compared to an annual expected change of approximately −0.108%. In contrast, the percent change in the right hippocampus did not significantly differ from the expected value, with *t*(16) = −1.559 and *p* = 0.139. The average percent change in the right hippocampus was −1.062% (SD = 2.522). These findings indicate a lateralized effect, with the left hippocampus showing significantly greater volume decline than would be expected based on healthy aging alone (see Figure 2C).

## 4. Discussion

The present study offers novel insight into the structural plasticity of the hippocampus in response to long-duration spaceflight, revealing significant volumetric changes with notable lateralization and sex-specific patterns. Building on the existing literature that identifies the hippocampus as sensitive to environmental stressors, our findings extend this understanding into the unique context of spaceflight. In the subsequent discussion, we explore the potential mechanisms underlying these changes, interpret their functional significance, and contextualize our findings within both the past literature and broader implications for astronaut health and mission performance.

First, the significant decrease in percent volume of the left hippocampus may be attributed to a range of factors associated with spaceflight, including (but not limited to) exposure to microgravity, radiation, isolation, and confinement (see Figure 2A,B). Although our study design is not able to disentangle the contribution of these different factors to hippocampal structural changes, one may speculate that these particular structural changes are indeed indicative of a physiological response to stress. In fact, previous studies have demonstrated that exposure to stressors can have a direct negative impact on hippocampal structural properties, particularly in the left hippocampus, which has been shown to exhibit greater volume reduction under stress conditions [22], indicating a high vulnerability to stress, in particular to stressors such as exposure to radiation [12] and isolation and confinement [19]. Our findings are consistent with this previous evidence given the significant amount of stress astronauts experience during the entire duration of a spaceflight mission [33].

Furthermore, we found distinct differences in the hippocampal subregions. Specifically, our analyses revealed a significant percent volume decrease in the left anterior subregion and right body subregion (see Figure 2A,B). These findings are aligned with the evidence reported in previous studies showing that hippocampal subregions respond differently to exposure to stress [34] and aging [31]; in these studies, the anterior subregion of the hippocampus is reported to be more susceptible to stress (often resulting in greater volume loss), and the midsection/body transitional subregion is reported to be more susceptible to aging [35]. Although only the left anterior subregion and the right body subregion reached statistical significance in volume loss, it is relevant to note that both the left body and right anterior subregions showed a trend toward significance (*p* = 0.108 and *p* = 0.184, respectively), whereas the posterior subregions in both the left and right hippocampus were far from it (*p* = 0.667 and *p* = 0.875, respectively). Overall, our findings are consistent with a vulnerability of the anterior subregions of the hippocampus that could be due to stress exposure [36], and a susceptibility of the hippocampal midsection/body subregions that is consistent with an accelerated physiological aging process, which is well documented in astronauts following a spaceflight to the ISS [8,37].

In addition, with respect to functional specialization of hippocampal subregions, the anterior hippocampus is closely involved in emotional and episodic memory encoding, particularly through its connections with the amygdala, which contributes to the processing of emotionally significant memories [38]. In contrast, the body of the hippocampus acts as a transition zone, integrating spatial and contextual information to support broader memory functions [38,39], with the posterior subregion primarily responsible for the retrieval of spatial information relevant to navigation [35]. Structural alterations in the hippocampus may also contribute to changes in extended neural networks and cognitive strategies that astronauts may demonstrate while adapting to the new microgravity environment [37]. Therefore, given the significant relevance of hippocampal functions for effective cognitive performance, future studies may need to evaluate the extent to which the structural changes that we have documented in this study would impact the performance of important tasks critical during a space mission.

These region-specific vulnerabilities not only are structurally significant but may also carry functional implications, particularly given the known lateralization of hippocampal involvement in distinct cognitive processes [34,40]. In fact, on the functional lateralization of the hippocampus, there is convincing evidence in the literature supporting the general role of the left hippocampus in verbal memory processing [41] and the right hippocampus’s greater involvement in spatial processing and navigational memory [40].

To further support the specificity of these effects, we compared the hippocampus to adjacent and functionally distinct control regions, finding nonsignificant changes in percent volume change. Our relevant finding of a significant decrease in the left hippocampus, in tandem with the analyses of our control regions showing no significant changes for this time period, provides an understanding of the regional specificity of hippocampal volumetric changes. For context, the amygdala was selected as the location control to account for changes in a structure within the medial temporal lobe that is adjacent to the hippocampus [30], and the caudate nucleus was selected as the functional control given its involvement in procedural memory [31].

To delineate the role of spaceflight-specific stressors in inducing the changes observed, we supplemented these findings with our natural aging model, which confirmed the significant findings for the left hippocampus, suggesting that certain regions of the brain may be particularly susceptible to the cumulative stressors of long-duration space travel, such as microgravity, radiation, and social isolation. By confirming that the left hippocampal atrophy in astronauts significantly exceeded normative age-related decline, our data suggest that these changes are not simply a result of natural aging processes. Rather, they point to a spaceflight-specific effect, especially for the left hemisphere.

Interestingly, previous studies in non-astronauts have also reported sex differences in hippocampal volume changes under stress, wherein males often experience a greater volume loss than females [33], which is consistent with our data revealing a greater volume loss in the right hippocampus of male astronauts as compared to female astronauts. Our finding of a significant sex difference in right hippocampal volume change—where male astronauts exhibited greater volume loss—may have functional implications for spatial cognition. Prior research demonstrated that males typically have a larger gray matter volume in the right anterior hippocampus [36,42], a region closely associated with three-dimensional mental rotation and spatial visualization performance. Moreover, the study showed that this anatomical difference accounted for much of the observed sex difference in spatial task performance. In the context of spaceflight, where spatial orientation and navigation are critical, a disproportionate volume loss in this region among male astronauts could reflect loss from a higher baseline or a heightened susceptibility to spaceflight-related stressors such as microgravity or radiation. These findings suggest the need to further explore how baseline hippocampal anatomy may interact with spaceflight-induced changes, and whether sex-specific structural vulnerabilities contribute to differential cognitive outcomes during or after missions.

It is important to address an apparent discrepancy in our findings: while we observed a significant sex-based difference in volume change in the whole right hippocampus, this difference did not reach statistical significance at the subregion level. This may appear counterintuitive, as one might expect that a significant change in the whole structure would be mirrored by at least one of its subregions. However, this can be explained by a few considerations. First, the whole-hippocampus analysis aggregates volume changes across all subregions, effectively increasing the statistical power to detect a global effect that may be distributed modestly but consistently across multiple subregions. In contrast, subregion-specific analyses are based on smaller absolute volume differences and more restricted variance, which can limit the detection of statistically significant changes, particularly in studies with small sample sizes like ours. Additionally, trends toward significance were observed in some subregions (e.g., right anterior and right body; *p* = 0.093 and *p* = 0.087, respectively), suggesting that while no single subregion exhibited a strong enough effect to reach significance independently, the cumulative volume loss across subregions likely contributed to the significant whole-hippocampus difference. These findings highlight the importance of interpreting regional effects within the broader context of whole-structure changes and underscore the challenges of statistical sensitivity in small-sample subregional analyses.

Past studies on microgravity-induced fluid redistribution during long-duration spaceflight highlight a concern in the interpretation of volumetric and structural changes [8]. While such studies offer important insights into brain-wide structural and fluid distribution changes associated with spaceflight, their scope and methodological focus differ significantly from our targeted analysis of hippocampal volume. Specifically, the study used diffusion imaging and macrostructural volume estimates to assess global brain changes—including ventricular expansion and cerebrospinal fluid shifts—without a region-specific focus on the hippocampus using high-resolution anatomical protocols [8]. The study emphasized broad associations between fluid redistribution and brain morphology, but it did not isolate the hippocampus using protocols such as the Harmonized Hippocampal Protocol [29], which our study employed to increase precision and reproducibility. Our analysis also used a location control (the amygdala) and a functional control (the caudate nucleus), both of which would also likely be influenced by generalized intracranial pressure shifts if fluid redistribution were a significant confounding factor. The absence of significant volume changes in these control regions supports the specificity of our findings in the hippocampus. Finally, our study design did not rely on indirect proxies of fluid dynamics (e.g., ventricle size) but instead focused on pre- and post-flight imaging comparisons using the Harmonized Hippocampal Protocol, which enhances anatomical accuracy and interpretive validity. As such, while we acknowledge the importance of fluid shifts in space neuroscience, our results reflect hippocampus-specific structural changes that are not attributable solely to ventricular expansion.

Despite our compelling preliminary findings, our study has the typical limitations encountered in space health research in astronauts [3], which are a small sample size and lack of a control group, which limit our ability to draw definitive conclusions on the specific factors contributing to hippocampal volume changes. While a large sample of participants could soon be feasible given the prospect of private space missions, a matched-groups design would strengthen our findings by allowing a direct comparison between astronauts undergoing a space mission and astronauts with similar demographic characteristics and experiences who did not undergo a spaceflight. In addition, while we hypothesize that stressors such as microgravity, radiation, and isolation are driving the observed changes, we cannot conclusively identify which factors were responsible, nor their relative contributions; future studies may be able to address this limitation by disentangling the complex interaction between the many stressors impacting astronauts’ neurophysiology.

Future work should build on these findings by more deeply examining sex- and hemisphere-specific vulnerabilities to spaceflight-induced brain changes, particularly given our observation of differential volume loss in the right hippocampus among male astronauts. Parsing out whether these effects reflect pre-existing anatomical differences, differential susceptibility to spaceflight stressors, or both will be critical to understanding individualized neurocognitive risk profiles. Expanding analyses to other brain regions, such as the hypothalamus, which plays a central role in stress regulation, hormonal feedback, and circadian rhythms, may offer further insight into the systemic physiological adaptations occurring during long-duration missions. To bridge structure and function, future studies should incorporate functional MRI (fMRI) and diffusion imaging (DTI) to assess whether structural changes translate into altered functional connectivity or white matter integrity, particularly in networks involving memory, navigation, and emotional regulation. Coupling these neuroimaging approaches with behavioral cognitive testing—especially tasks assessing spatial reasoning, memory encoding, and stress resilience—may help determine the practical implications of the brain changes observed and guide targeted interventions to mitigate risk for future astronauts. Ultimately, longitudinal, multimodal, and demographically representative research will be essential for advancing our understanding of the effects of spaceflight on the health of astronauts.

## 5. Conclusions

In this study, we identified significant volumetric changes in the hippocampus of astronauts following a six-month mission aboard the International Space Station. These changes were regionally specific, lateralized to the left hemisphere, and more pronounced in male astronauts. These findings provide the very first direct evidence of hippocampal volumetric changes in astronauts following a long-duration spaceflight, highlighting the hippocampus as a key neural structure affected by space-related stressors. High-resolution, subregion-specific analyses such as ours may be of practical use in monitoring astronaut brain health and informing countermeasures to safeguard cognitive performance during future missions.

## Data Availability

The data presented in this study are available on request from the corresponding author due to astronauts’ privacy protection.

## Supplementary Materials

The following supporting information can be downloaded at https://www.mdpi.com/article/10.3390/neurosci6030070/s1, Figure S1: Percent volume change in control regions as analyzed by a one-sample *t*-test (A,B). Hippocampal subregions percent volume changes stratified by sex and analyzed with independent-samples *t*-tests with sex as a grouping variable (C,D).

## Abbreviations

The following abbreviations are used in this manuscript:

ISS	International Space Station
MRI	Magnetic Resonance Imaging
CSA	Canadian Space Agency
MPRAGE	Magnetization-Prepared Rapid Gradient Echo
FLAIR	Fluid-Attenuated Inversion Recovery
