# Supplementary material for: Hippocampal Volumetric Changes in Astronauts Following a Mission in the International Space Station
Source: NeuroSci. 2025 Jul 25;6(3):70. doi: 10.3390/neurosci6030070 (PMC12372098; doi:10.3390/neurosci6030070)
Supplement: Supplementary file 1 [file neurosci-06-00070-s001.zip › neurosci-3708243-supplementary.pdf]

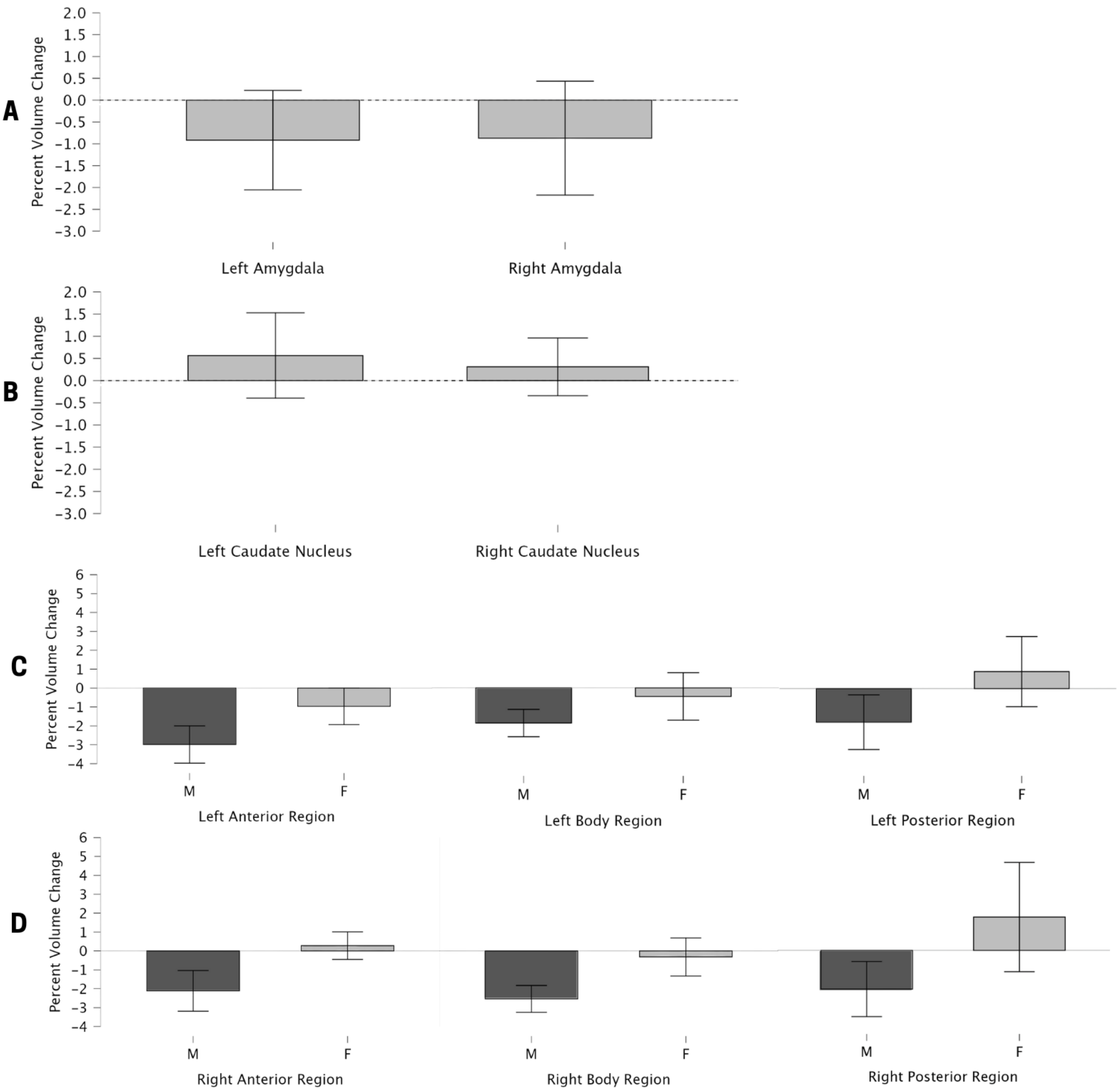

Figure S1.
Percent volume change in control regions as analyzed by a one-sample t-test (Panels A and B). Hippocampal subregions percent volume changes stratified by sex and analyzed with independent-samples t-tests with sex as a grouping variable (Panels C and D).
(A) Percent volume change in the left and right amygdala, included as anatomical control regions adjacent to the hippocampus, showed no significant differences ($p = 0.433$ and $p = 0.514$, respectively).
(B) Percent volume change in the left and right caudate nucleus, included as functional control regions involved in procedural memory, also revealed no significant change ($p = 0.566$ and $p = 0.640$, respectively). Error bars indicate standard error of the mean.
(C) Percent volume change in the left anterior, body, and posterior hippocampal subregions for males (dark grey) and females (light grey), analyzed with independent-samples t-tests with sex as a grouping variable.
(D) Percent volume change in the right anterior, body, and posterior hippocampal subregions for males and females. A wider y-axis range is shown in Panels C and D to reflect the higher variability in percent volume change due to smaller sample sizes when stratifying by sex. No significant sex-based differences were found in any subregion (left anterior: $p = 0.167$; right anterior: $p = 0.093$; left body: $p = 0.334$; right body: $p = 0.087$; left posterior: $p = 0.268$; right posterior: $p = 0.242$).
